# In Silico, In Vitro, and In Vivo Antinociceptive Potential of *S*‐Naproxen Derivatives

**DOI:** 10.1002/open.202500493

**Published:** 2026-03-27

**Authors:** Ubaidullah, Naveed Muhammad, Abad Khan, Rahaf Ajaj, Abdur Rauf, Umer Rashid, Fahad Hussain, Rashid Khan, Faiza Seraj, Uzma Uzma, Basharat Ali, Abdul Sadiq, Khalid Mohammed Khan, Marcello Iriti

**Affiliations:** ^1^ Department of Pharmacy University of Swabi Swabi Khyber Pakhtunkhwa Pakistan; ^2^ Department of Pharmacy Abdul Wali Khan University Mardan Khyber Pakhtunkhwa Pakistan; ^3^ College of Engineering Abu Dhabi University Abu Dhabi United Arab Emirates; ^4^ Research Institute for Sustainable Futures Abu Dhabi University Abu Dhabi United Arab Emirates; ^5^ Department of Chemistry University of Swabi Anbar Khyber Pakhtunkhwa Pakistan; ^6^ Deparment of Pharmacy COMSAT University Abbottabad Abbottabad Pakistan; ^7^ H. E. J. Research Institute of Chemistry International Center for chemical and Biological Sciences University of Karachi Karachi Pakistan; ^8^ Sulaiman Bin Abdullah Aba Al‐Khail Center for Interdisciplinary Research in Basic Science International Islamic University Islamabad Pakistan; ^9^ Department of Pharmacy University of Malakand Chakdara Pakistan; ^10^ Pakistan Academy of Sciences Islamabad Pakistan; ^11^ Department of Biomedical, Surgical and Dental Sciences University of Milan Milan Italy; ^12^ National Interuniversity Consortium of Materials Science and Technology (INSTM) Firenze Italy

**Keywords:** acetic acid, COX, hot plate, molecular simulations, *S*‐naproxen derivatives

## Abstract

Selected naproxen derivatives were investigated for their antinociceptive effects, using in vitro (COX‐I/COX‐II antagonistic effect), in vivo (acetic acid and hot plate assay), and in silico (molecular simulation studies) approaches. The COX inhibition studies revealed that the maximum percent effect exhibited by **GMO‐I‐111a** 96.01 and 92% against COX‐2 and COX‐1, respectively, at higher concentrations. The effect at minimum concentration was 81 and 77% against COX‐1 and COX‐2, respectively. The COX antagonist effect of **GMO‐I‐9a** and **GMO‐I‐11a** was also outstanding in the acetic acid assay. All the tested derivatives demonstrated a significant (*p* < 0.05) analgesic effect at all concentrations (1.25, 2.5, and 10 mg/kg). The maximum effect was found against **GMO‐I‐135a** (95%), followed by **GMO‐I‐11a** (92%). The central analgesic effect of various derivatives was significant (*p* < 00.1). **GMO‐I‐111a** exhibited a significant (*p* < 00.1) analgesic effect after 30 min at all the tested doses. The maximum effect at higher doses was shown by compound **GMO‐I‐9a** (93%) followed by **GMO‐I‐11a** (88%), **GMO‐I‐111a** (84%), and **GMO‐I‐135a** (78%). The latency time of **GMO‐I‐9a** and **GMO‐I‐135a** was significantly reversed by naloxone treatment, indicating the central analgesic effect. The molecular simulation studies showed that all the tested compounds had good binding interactions with COX‐1/COX‐2, except **GMO‐I‐9a**. In conclusion, the tested naproxen derivatives might have a significant analgesic effect.

## 1 Introduction

Propionic acid is one of the simple and smart carboxylates that is normally found in fibrous food as well as in milk products. This fibrous food is helpful in the management of decreasing body weight and diabetes [1]. These physiological aspects of fiber‐containing food are attributed to metabolites such as short‐chain fatty acids (acetate, propionate, and butylate) [2]. These metabolites are the result of the digestion of the mentioned food materials. The primary source of indigenous propionic acid (PA) is propionibacterium's fermentation of dietary fibers [3]. The PA is an energy source as well as having many beneficial biological aspects such as reduction in low‐grade intestinal inflammation [4] and inhibition of cyclooxygenase (COX) [5]. The COX is notorious for the production of oxygenated eicosanoids (proinflammatory). Due to the antagonistic effect of COX, several PA derivatives were synthesized, including ibuprofen, ketoprofen, dexibuprofen, and naproxen [6]. The PA is optically inactive; however, when one of the hydrogens of carbon number two is replaced by any group or branch, carbon 2 demonstrates optical activity.

Based on the chirality of carbon 2, the analgesic, inflammatory, and antipyretic effect is changing, as the *S*‐naproxen is pharmacologically active. At the same time, the *R*‐naproxen is devoid of these pharmacological actions. That is why all naproxen derivatives in the current studies have levorotatory stereochemistry. Naproxen was introduced in 1976 and accepted by the FDA as over‐the‐counter (OTC) in 1994. Due to the acceptance of naproxen as OTC, it became the most selling analgesic, antiinflammatory, and antipyretic drug [7]. Naproxen is a well‐known nonsteroidal antiinflammatory (NSAID) drug used for the treatment or management of various painful conditions. Different studies confirmed the potency and efficacy of naproxen more than available NSAIDs. The effect of this painkiller is significant in various rheumatic conditions as well as in primary and secondary dysmenorrhea. It is also reported that naproxen decreases excessive bleeding in menorrhagia patients [8]. The advantage of naproxen is its rapid absorption and long‐duration action with a half‐life of 13 h. The distribution of naproxen to synovial fluid is greater; therefore, it is the drug of choice in osteoarthritis patients prone to heart problems because naproxen has a lower risk of vascular problem induction than other NSAIDs [9]. The current studies aim to develop safe and effective antinociceptive molecules based on *S*‐naproxen. It has been reported in the literature that various derivatives of naproxen were investigated with a low risk of gastric bleeding and hypertension as compared to the parent drug [10]. Different studies reported various pharmacological potentials of naproxen derivatives such as antioxidant, anticancer [11], antimicrobial [12], analgesic, anti‐inflammatory [13], urease inhibitory effects [14], and anti‐SARS CoV‐2 [15]. Considering these literature data, the current study was conducted to find safe, effective, and economical NSAIDs.

## 2 Materials and Methods

All the reagents and chemicals used were of analytical grade. Diclofenac sodium (Indus Pharmaceutical Pvt. Limited), tramadol (Seral Pharmaceutical Pvt. Limited), normal saline (Otsuka Pharmaceutical Pvt. Limited), indomethacin (Chines Pharmaceuticals), celecoxib (Getz Pharmaceutical Pvt. Limited), and rest of reagents were purchased from Sigma Aldrich. Prof. Dr. Khalid Mohammed Khan, H. E. J. Research Institute of Chemistry, International Center for Chemical and Biological Sciences, University of Karachi, Karachi‐75,270, Pakistan, provided the compounds to be tested. The detail of these compounds is presented in Table 1 [14].

**TABLE 1:** List of tested different naproxen derivatives.

S. No.	Codes	Structure	Mol. formula	IPUC name
1	**GMO‐I‐9a**	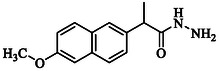	C_14_H_16_N_2_O_2_	2‐(6‐Methoxynaphthalen‐2‐yl)propanehydrazide
2	**GMO‐I‐11a**	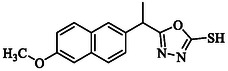	C_15_H_14_N_2_O_2_S	5‐(1‐(6‐Methoxynaphthalen‐2‐yl)ethyl)−1,3,4‐oxadiazole‐2‐thiol
3	**GMO‐I‐103a**	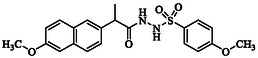	C_21_H_22_N_2_O_5_S	4‐Methoxy‐*N’*‐(2‐(6‐methoxynaphthalen‐2‐yl)propanoyl)benzenesulfonohydrazide
4	**GMO‐I‐111a**	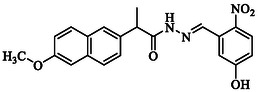	C_21_H_19_N_3_O_5_	*N′*‐(5‐Hydroxy‐2‐nitrobenzylidene)‐2‐(6‐methoxynaphthalen‐2‐yl)propanehydrazide
5	**GMO‐I‐135a**	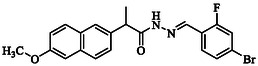	C_21_H_18_BrFN_2_O_2_	*N′*‐(4‐Bromo‐2‐fluorobenzylidene)‐2‐(6‐methoxynaphthalen‐2‐yl)propanehydrazide

### 2.1 Animals

In all the experiments, BALB/C mice of either sex were used, which were purchased from the National Institute of Health (N.I.H) Islamabad Pakistan Pharmacology Section. The animals were fed with standard food and water *ad libitum* and were maintained in the standard laboratory conditions (25°C and light/dark cycles 12 h). The Department of Pharmacy Abdul Wali Khan University Mardan Pakistan Ethical Committee approved the experimental protocols of the animals.

### 2.2 Acute Toxicity

The selected naproxen derivatives were tested for acute toxicity studies at various doses. The animals were classified into different groups, i.e., negative control and tested groups. The naproxen was injected IP, and the individual animals were observed for 4 h for drinking, feeding, walking, hair, ear erection, etc. The mortality rate was recorded after 24 h [16].

### 2.3 Cyclooxygenase (COX‐I/COX‐II) Assay

The COX‐I/II assay of selected naproxen derivatives was conducted using the published procedure with some modifications. Different concentrations of naproxen derivatives were prepared. COX‐I (conc. 0.7–0.8 g/L) was used for sheep sources, and COX‐II (conc. of 300 units/mL) was used for human recombinant sources. The substrate for these enzymes was used as arachidonic acid and linolenic acid. The COX‐II solution was made ready for up to 300 units/mL. Before adding 50 µL co‐factor solution, 10 µL of enzyme solution was kept on ice for 5 min. The solution is a mixture of 1 mM hematin in a 0.1 M tris buffer with pH 8, 0.24 mM TMPD, and 0.9 mM glutathione. The enzyme and experimental solutions were kept at normal temperature for 5–10 min. Arachidonic acid was used to start the procedure, and the mixture was kept at 37°C for 15 min. The UV visible spectrophotometer was used to find the absorbance level, and the percent effect was calculated [17].

### 2.4 Acetic Acid‐Induced Writhing

The naproxen derivatives were evaluated for analgesic effect using acetic acid‐induced writhing test [16]. The animals were classified as negative control (DW, 10 mL/kg), positive control (diclofenac sodium, 10 mg/kg), and tested groups (1.25, 2.5, and 5 mg/kg). After 30 min of the above treatment, each animal was injected with a 1% acetic acid (10 mL/kg) solution to induce abdominal contractions (writhes). The number of writhings was counted for 10 min, and the percent analgesic effect was calculated.

$$\%\text{analgesic effect}=100-\frac{\text{Number of writhings in tested animals}}{\text{Number of writings in control animals}}\times 100$$

### 2.5 Hot Plate Test

The thermal‐induced pain model evaluated analgesic effects at the above‐mentioned doses. The animals were classified as above. The negative control was treated with DW (10 mL/kg), the positive control was treated with tramadol (5 mg/kg), and the tested groups were naproxen derivatives (1.25, 2.5, and 5 mg/kg). The analgesiometer temperature was set at 55 ± 1°C. All the animals were pretested for latency time. Animals with more than 15 s latency on a hot surface were excluded from the study. After 30 min of all administration, the individual animal was placed on the heated surface, and the time duration without jumping or licking/flicking was recorded (latency time). The cutoff time was set as 30 s to avoid tissue damage. To know the involvement of opioidergic receptors, naloxone (0.5 mg/kg, SC) was used as above. The percent effect was calculated using the following formula [16].

$$\mathrm{Percent}\;\mathrm{effect}=\frac{\mathrm{Lt}-\mathrm{Lc}}{\mathrm{COT}-\mathrm{Lc}}\times 100$$

where Lt = Latency time of tested compounds, Lc = Latency time of control, and COT = cutoff time.

### 2.6 Muscle Coordination

#### 2.6.1 Inclined Plane

This experiment was performed according to our published protocol [18]. Here, the two plywood boards were used so that one board forms the base and the other is connected to form an angle of 65°. Then, the animals were classified as above; the positive control group was treated with diazepam (0.5 mg/kg), and the rest were administered as above. After 30 min of administration, the animal was placed on the upper part of the plywood for a 30‐second to check that the animal would slide or stay within the given duration.

#### 2.6.2 Traction Test

A rubber‐coated wire was used in this procedure. Both ends were fixed about 60 cm above the ground. The animals were classified above; the positive control was treated with diazepam (0.5 mg/kg), and the rest of the groups were treated as above. The animals were hanged for 5 s. The hanging animals were declared as having no muscle relaxant, and the failure was considered as relaxed muscle [18].

#### 2.6.3 Docking Study Methodology

To investigate the in vitro studies, docking simulation studies of COX‐1 and COX‐2 were carried out. The docking on targets was utilized on Molecular Operating Environment (MOE) software. The *S*‐naproxen derivatives were docked into the active site of COX‐1 (PDB id 1EQG) and COX‐2 (1CX2). The crystallographic structure of proteins was accessed from the Protein Data Base (PDB). Their PDB ID is 1EQG for COX‐1 and 1CX2 for COX‐2. The previously reported methods were used to prepare 3‐D structures of naproxen (*S*) derivatives and the downloaded enzymes. The previously reported methods were used to prepare the 3‐D structures of *S*‐naproxen derivatives and the enzymes (COX‐1 and COX‐2). MOE was used to dock the compounds into the binding site where the cocrystallized ligand is present. The retrieved structure of a protein is firstly 3D protonated, and after that, its minimization is carried out at 0.1 gradients using the (force‐field) Amber10EHT in MOE (2016). All S‐naproxen derivative structures were prepared using the Molecular Builder Module Program in MOE. The structures were minimized at 0.00001 Gradient using the Amber10EHT force field. In the binding site of the protein, the compounds were docked, and multiple protein and ligand complex conformations were formed using the Triangular Matcher docking method. The ligands were ranked according to the scores of GBVI/WSA calculation. The GBVI/WSA is a scoring method that determines the free binding energy of a ligand from a certain location. Lower scores represent the more favorable pose among all scoring functions. Kcal/mol is the unit for this scoring method. The binding affinities of different protein‐ligand conformations (poses) were studied with the help of MOE and Discovery Studio.

#### 2.6.4 Statistical Analysis

The data were analyzed using GraphPad Prism. In all the experimental data, related statistical tests were performed to confirm the level of significance in comparison with the negative control.

## 3 Results

### 3.1 Design Rationale

The rationale behind the design of targeted naproxen‐based compounds was driven further by the recognized biochemical mechanism of action of naproxen as a cyclooxygenase enzyme inhibitor, where it exerts anti‐inflammatory, analgesic, and antipyretic effects through selective inhibition of COX‐1 and COX‐2 enzymes, resulting in reduction of PGSs synthesis. In this regard, naproxen, being a carboxylic acid‐containing compound, involves non‐shallowing with the COX enzyme catalytic channel through the recognition of the aryl group via hydrophobic recognition and dipolar interaction of the carboxylic acid moiety with important amino functionalities lining the catalytic channel of COX‐1 and COX‐2 enzymes. In this study, it can be noted that naproxen as an analgesic was maintained, considering its recognized activity as a COX enzyme inhibitor, with an attempt to elevate further the activity through design of naproxen‐based drugs containing hydrazide‐hydrazone moieties as replacements for carboxylic functionalities, with an attempt to anchor further to increase activity recognition via hydrogen bonding through increased ligand‐enzyme activity. In this way, there was design of drugs with potential to elevate activity over naproxen as an analgesic through biochemical inhibition of COX‐1 and COX‐2 enzymes and, as such, elevate anti‐nociceptive activity.

### 3.2 Effect on COX

The effect of tested compounds on COX 1/2 is presented in Table 2. The selected derivatives were tested at different concentrations (500, 250, 125, 62.5, 31.25 µg/mL). The maximum inhibition was noted against **GMO‐I‐111a** exhibited 96.01 (COX‐2) and 92% (COX‐1) effects at higher concentrations. The percent effect at minimum concentration was 81 and 77 against COX‐1 and COX‐2, respectively. The maximum antagonistic effect of **GMO‐I‐111a** was demonstrated by **GMO‐I‐9a** with a percent effect of 89 and 76 against COX‐1 and COX‐2, respectively. The inhibitory effect of **GMO‐I‐11a** was also outstanding.

**TABLE 2:** Results of in‐vitro inhibitory potentials of compounds against COX‐1/COX‐2 enzymes.

Codes	**Conc.,** **µg/mL**	**% COX‐1 inhibition** **(mean ± SEM)**	**% COX‐2 inhibition** **(mean ± SEM)**
**GMO‐I‐9a**	500 250 125 62.5 31.25	76.87 ± 0.32 74.63 ± 1.88 71.08 ± 0.99 65.96 ± 0.35 62.35 ± 0.85	89.36 ± 1.05 86.96 ± 1.53 83.63 ± 1.93 79.30 ± 1.00 71.52 ± 0.96
**GMO‐I‐135a**	500 250 125 62.5 31.25	70.69 ± 1.01 66.63 ± 2.00 59.50 ± 0.22 57.63 ± 1.66 54.89 ± 0.88	77.01 ± 1.03 73.63 ± 0.31 66.52 ± 1.85 60.85 ± 0.27 52.35 ± 1.00
**GMO‐I‐103a**	500 250 125 62.5 31.25	72.05 ±1.05 66.52 ± 2.03 62.05 ± 1.28 53.87 ± 0.68 48.62 ± 2.05	81.02 ± 1.13 76.53 ± 2.50 71.85 ± 0.22 66.85 ± 0.89 57.52 ± 1.96
**GMO‐I‐11a**	500 250 125 62.5 31.25	81.32 ± 0.52 73.96 ± 3.63 69.52 ± 3.20 56.50 ±1.00 39.50 ± 2.12	84.03 ± 0.66 79.52 ± 2.65 77.62 ± 0.96 72.05 ±2.65 61.44 ± 2.00
**GMO‐I‐111a**	500 250 125 62.5 31.25	92.05 ± 2.05 91.36 ± 0.96 86.86 ±1.11 83.52 ± 0.85 81.08 ±1.25	96.01 ± 1.33 91.52 ± 2.56 87.06 ±2.78 84.96 ±1.56 77.52 ± 3.65
Indomethacin	500 250 125 62.5 31.25	97.02 ± 0.05 94.62 ± 1.06 91.05 ± 1.55 86.63 ± 2.02 81.05 ±1.05	—
Celecoxib	500 250 125 62.5 31.25	—	95.63 ± 1.25 92.30 ± 1.05 88.63 ± 1.36 86.65 ± 2.07 79.88 ± 1.55

### 3.3 Acute Toxicity

The acute toxicity of selected naproxen derivatives and clinical scoring are presented in Table 3. The selected derivatives were tested at higher doses, i.e., 10 and 15 mg/kg IP. The animals were observed for consecutive 4 h to note the clinical scoring such as muscle relaxation, sedation, feces, and ear erection. At the same time, the mortality rate was recorded for up to 24 h. During 4 h acute assessment, it was noted that animals treated with both tested doses were found with less frequency of micturition and defecation reflex. The drinking and feeding behavior was also disturbed; however, animals had free access to water and food *ad labitum.* The hairs and ear erection were noted with disturbed behavior.

**TABLE 3:** Acute toxicity studies of naproxen derivatives.

Compounds	Doses	Feces	Food	Urination	Drinking	Ear erection	Hair erection	Mortality
**GMO‐I‐9a**	2.5	−	−	−	−	−	−	−
	5	−	−	−	−	−	−	−
	10	less	–	less	less	+	+	–
**GMO‐I‐11a**	15	less	–	less	less	+	+	–
	2.5	–	–	–	–	–	–	–
	5	–	–	–	–	–	–	–
**GMO‐I‐103a**	10	less	–	less	less	+	–	–
	15	less	–	less	less	+	–	–
	2.5	–	–	–	–	–	–	–
**GMO‐I‐111a**	5	–	–	–	–	–	–	–
	10	less	–	less	less	+	+	–
	15	less	–	less	less	+	+	–
**GMO‐I‐135a**	2.5	–	–	–	–	–	–	–
	5	–	–	–	–	–	–	–
	10	less	–	less	less	+	+	–
	15	less	–	less	less	+	+	–

### 3.4 Effect of Acetic Acid‐Induced Writhing

**FIGURE 1:**
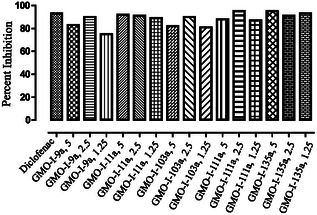
Percent effect of naproxen derivatives in acetic acid‐induced writhing model.

The analgesic effect of various naproxen derivatives in acetic acid‐induced writhing is presented in Table 4. All of the tested naproxen derivatives demonstrated a significant (*p* < 0.001) analgesic effect at all the tested doses (1.25, 2.5, 5, and 10 mg/kg). The induced writhings were significantly attenuated by all tested doses. The maximum percent analgesic effect was found against **GMO‐I‐135a** (95%) followed by **GMO‐I‐11a** (92%) as shown in Figure 1.

**TABLE 4:** Analgesic effect of different naproxen derivatives in acetic acid‐induced writhing model.

Compounds	Dose, mg/kg	Writhing's
**D/W**	10 mL	70.55 ± 0.98
**Diclofenac**	10	4.75 ± 0.23***
**GMO‐I‐9a**	5	11.87 ± 1.98***
	2.5	6.87 ± 0.38***
	1.25	17.45 ± 0.91***
**GMO‐I‐11a**	5	5.41 ± 0.91***
	2.5	6.12 ± 1.98***
	1.25	7.34 ± 0.92***
**GMO‐I‐103a**	5	12.67 ± 0.68***
	2.5	6.98 ± 0.91***
	1.25	12.72 ± 1.98***
**GMO‐I‐111a**	5	8.41±0.58***
	2.5	2.87±2.98***
	1.25	8.92±0.48***
**GMO‐I‐135a**	5	3.39±0.90***
	2.5	5.76±1.99***
	1.25	4.51±0.28***

Values are presented as mean ± SEM for group six animals. The data were analyzed by ANOVA, followed by Dunnett's test.

### 3.5 Hot Plate Test

**FIGURE 2:**
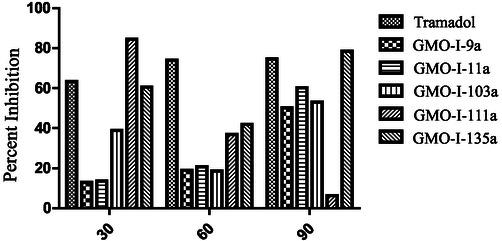
Percent analgesic effect of naproxen derivatives at 5 mg/kg.

The central analgesic effect of naproxen derivatives is demonstrated in Table 5. Various derivatives showed significant (*p* < 0.001) analgesic effects at different time intervals. The latency time was prolonged from 30 min of administration and remained significant up to the end of the experimental duration. The **GMO‐I‐111a** exhibited a significant (*p* < 0.01) analgesic effect after 30 min at all the tested doses, even up to the end of the experiment. The maximum percent effect at higher doses was shown by compound **GMO‐I‐9a** (93%) followed by **GMO‐I‐11a** (88%), **GMO‐I‐111a** (84%), and **GMO‐I‐135a** (78%), as shown in Figure 2. The positive control (tramadol) demonstrated maximum analgesic effect. Naloxone was used for the evaluation of the selected derivatives for the confirmation of the central effect. It was observed that the latency time of **GMO‐I‐9a** and **GMO‐I‐135a** was significantly reversed, indicating the central analgesic effect.

**TABLE 5:** Thermal‐induced analgesic effect of NPX derivatives.

Compounds	**Dose,** **mg/kg**	0 min	30 min	60 min	90 min
**D/W**	10 mL	6.21 ± 0.55	6.55 ± 0.82	5.90 ± 0.67	6.15 ± 0.72
**Tramadol**	5	6.80 ± 0.61	18.22 ± 0.71***	20.05 ± 0.59***	20.22 ± 0.59***
**GMO‐I‐9a**	5	6.42 ± 0.58	8.39 ± 0.54	9.52 ± 0.81	15.60 ± 0.49***
	2.5	6.03 ± 0.70	13.73 ± 0.74**	14.92 ± 0.69**	23.73 ± 0.85***
	1.25	6.28 ± 0.81	8.13 ± 0.65	7.25 ± 0.91	8.95 ± 0.79
**GMO‐I‐11a**	5	6.81 ± 0.59	25.65 ± 0.81***	9.85 ± 0.68	17.50 ± 0.82***
	2.5	6.01 ± 0.90	10.96 ± 0.75*	11.98 ± 0.80**	11.37 ± 0.42**
	1.25	7.01 ± 0.80	7.91 ± 0.63	11.80 ± 0.69**	14.50 ± 0.79***
**GMO‐‐I‐103a**	5	7.33 ± 0.82	13.73 ± 0.67**	9.45 ± 0.90*	16.14 ± 0.70***
	2.5	8 0.00 ± 0.75	11.55 ± 0.62**	13.56 ± 0.77**	19.08 ± 0.58***
	1.25	8.60 ± 0.68	11.55 ± 0.39**	6.48 ± 0.67	14.50 ± 0.70**
**GMO‐I‐111a**	5	6.95 ± 0.69	22.13 ± 0.82***	12.93 ± 0.87**	7.33 ± 0.62
	2.5	6.49 ± 0.73	12.96 ± 0.65**	13.58 ± 0.55**	14.69 ± 0.70**
	1.25	7.91 ± 0.90	14.90 ± 0.79**	14 0.05 ± 0.81**	21.02 ± 0.63***
**GMO‐I‐135a**	5	6.32 ± 0.78	17.72 ± 0.68***	13.90 ± 0.75**	20.92 ± 0.47***
	2.5	6.90 ± 0.59	7.90 ± 0.59	9.35 ± 0.44*	9.55 ± 0.81*
	1.25	5.80 ± 0.57	12.43 ± 0.94**	14.50 ± 0.87**	8.95 ± 0.58
**Naloxone treatment**					
**GMO‐I–I‐9a**	5	7.54 ± 0.43	6.82 ± 0.64	10.78 ± 0.64	11.0 ± 0.23
**GMO‐I‐11a**	5	12.00 ± 0.64	12.81 ± 0.77	12.02 ± 0.55	9.80 ± 0.76
**GMO‐I‐103a**	5	8.28 ± 0.87	10.91 ± 0.58	11.84 ± 0.76	8.45 ± 0.55
**GMO‐I‐111a**	5	9.81 ± 0.22	12.15 ± 0.43	10.02 ± 0.32	11.02 ± 0.58
**GMO‐I‐135a**	5	10.73 ± 0.96	7.90 ± 0.31	9.30 ± 0.97	9.00 ± 0.85

Values are presented as mean ± SEM for group six animals. The data were analyzed by ANOVA followed by Dunnett's test.

### 3.6 Muscle Coordination Effect

No muscle relaxant effect was noted for all tested compounds.

### 3.7 Docking Studies on COX‐1

**FIGURE 3:**
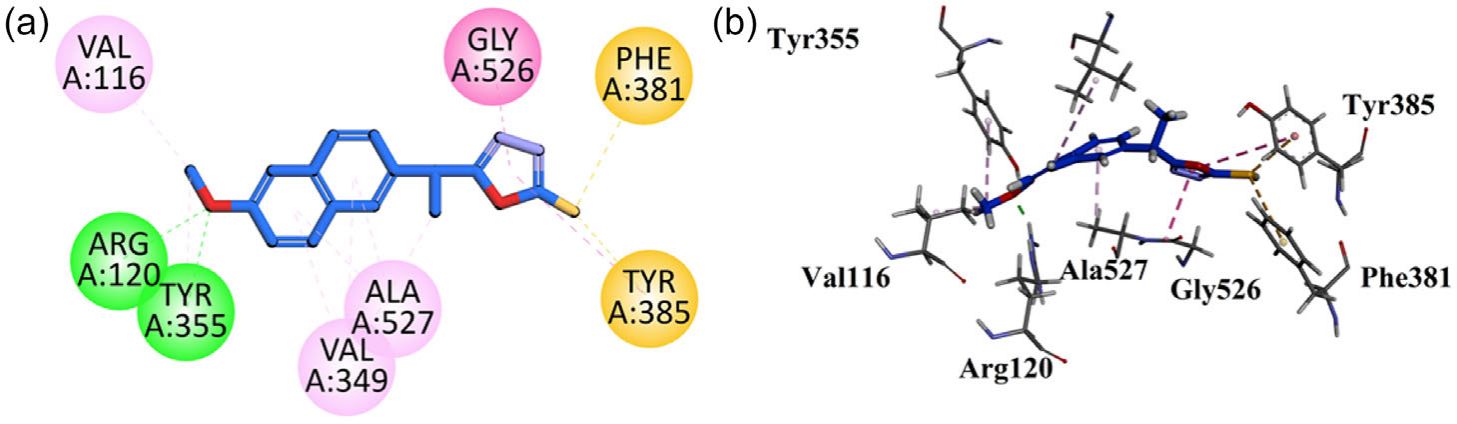
2D (a) and 3D (b) Plot of GMO‐I‐11a with COX‐1.

To investigate the binding affinities of *S*‐naproxen derivatives, they were docked against the COX‐1 active site. The native ligand binding interactions include Val116, Leu359, Tyr355, Aarg120, Val349, and Ala527. Compound **GMO‐I‐9a** does not display important interaction with the residues of the binding site. The compound **GMO‐I‐11a** exhibited two important hydrophilic interactions with amino acid residues (Arg120 and Tyr355) similar to the native ligand binding interactions. Other interaction includes π‐sulfur binding interaction with Phe381 and Tyr385 amino acid residues with the thiol region present at the terminal of the compound. Gly526 exhibited amide‐π staked interaction with the oxadiazole region of the compound. Other hydrophobic (π‐Alkyl) interactions include Val116, Val349, and Ala527 with the compound's aromatic and terminal methyl region (Figure 3).

**FIGURE 4:**
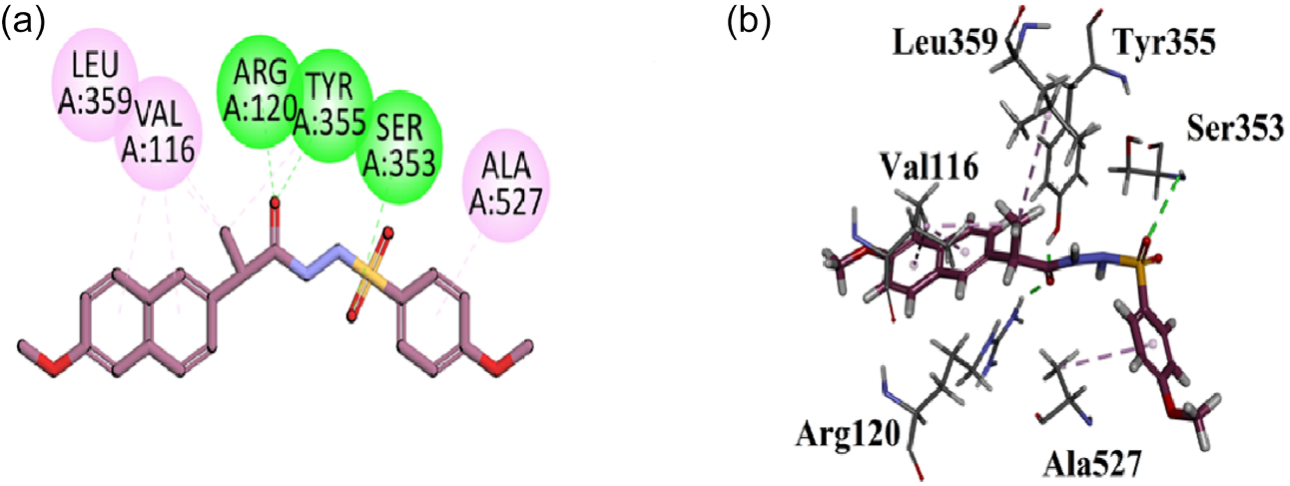
2D (a) and 3D (b) Plot of GMO‐I‐103a with COX‐1.

Compound **GMO‐I‐103a** exhibited three hydrophilic hydrogen bond interactions with the Arg120, Tyr355, and Ser353 through the carbonyl and sulfonyl oxygen regions. Other hydrophobic interaction includes Leu359, Val116, and Ala527. All six amino acid residues are also displayed as native ligands (Figure 4).

**FIGURE 5:**
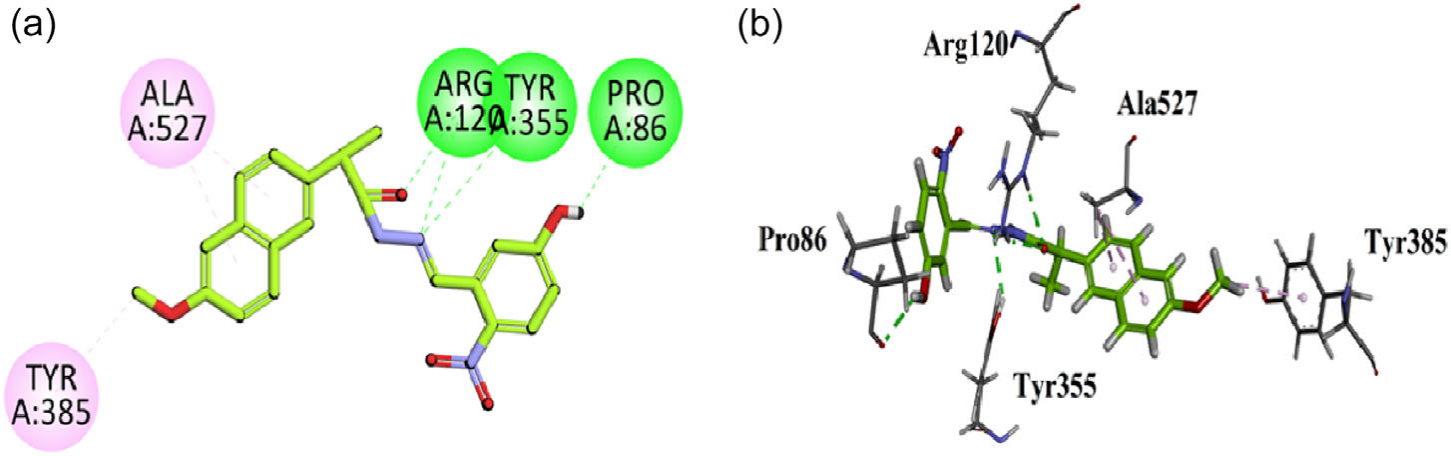
2D (a) and 3D (b) Plot of GMO‐I‐111a with COX‐1.

Compound **GMO‐I‐111a** exhibited hydrogen bond interaction with the Arg120, Tyr355, and Pro86. Other binding affinities include hydrophobic interaction with amino acid residues Ala527 and Tyr385 (Figure 5).

**FIGURE 6:**
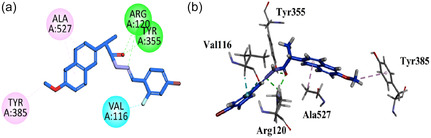
2D (a) and 3D (b) Plot of GMO‐I‐135a with COX‐1.

Compound **GMO‐I‐135a** displayed two important binding affinities (hydrophilic hydrogen bond interaction) with the Arg120 and Tyr355 through the amide linkage's carbonyl region and NH region. It also forms halogen interaction with the Val116 with the halogen attached at the aromatic region. On the other terminal of the molecule, hydrophobic interactions are exhibited by the Ala527 and Tyr385 amino acid residues (Figure 6).

### 3.8 Docking Studies on COX‐2

**FIGURE 7:**
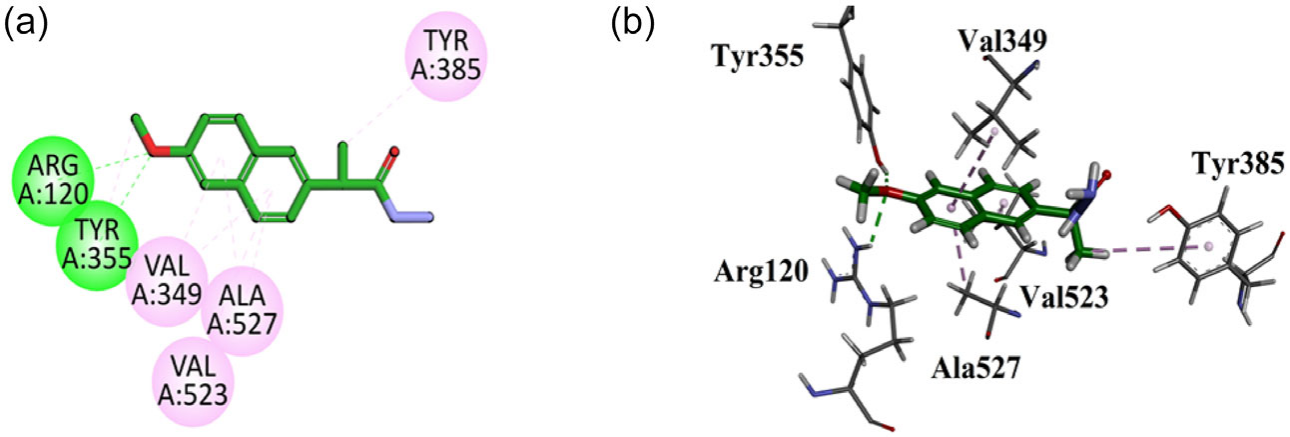
2D (a) and 3D. (b) Plot of GMO‐I‐9a with COX‐2.

The *S*‐naproxen derivatives were docked into the active site of COX‐2 (PBD ID = 1CX2) having S58 (selective inhibitor) to find the binding affinities using a molecular modeling study. The selective inhibitor of COX‐2 displayed interactions with important residues including hydrophilic binding affinities with Arg513, Leu352, Ser353, His90, Tyr355, Arg120, and hydrophobic interactions with Val349, Val523, Tyr385, Trp387, Phe381, Leu384, and Ala527. Compound **GMO‐I‐9a** displayed two important hydrogen bond binding affinities with important residues Arg120 and Tyr355 through the oxygen of the methoxy group attached to the aromatic region. Other binding affinities are hydrophobic interactions (Val349, Val523, Ala527, and Tyr385) with residues similar to the native ligand (Figure 7).

**FIGURE 8:**
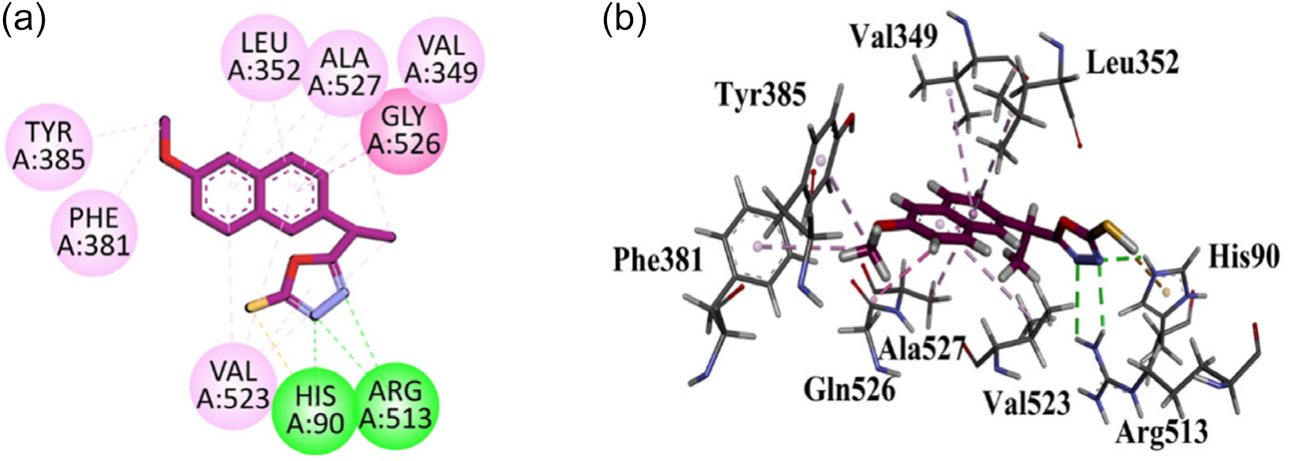
2D (a) and 3D (b) Plot of GMO‐I‐11a with COX‐2.

Compound **GMO‐I‐11a** exhibited two hydrogen bond hydrophilic interactions with His90 and Arg513. The His90 also displayed a π‐sulfur binding affinity with the thiol group present at the terminal of the compound. Gly526 also displayed π‐π stacked interaction with the aromatic region (Figure 8). Other hydrophobic interactions include (Tyr385, Phe381, Val523, Val349, Ala527, and Leu352).

**FIGURE 9:**
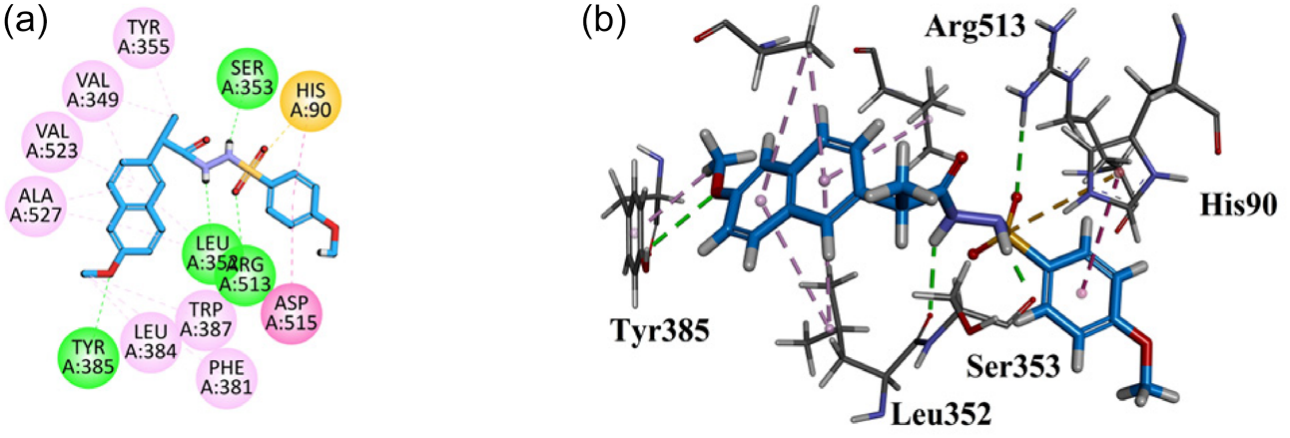
2D (a) and 3D (b) Plot of GMO‐I‐103a with COX‐2.

Compound **GMO‐I‐103a** exhibited 4 important hydrophilic hydrogen bond interactions with Ser353, Tyr385, Leu352, and Arg513. π‐Sulfur binding affinity and π‐π T‐shaped was displayed by His90. Asp515 exhibited π‐ π staked interaction with terminal aromatic moiety. Other hydrophobic π‐alkyl interactions (Tyr355, Val349, Val523, Ala527, Trp387, Leu384, and Phe381) include important residues similar to native ligand (Figure 9).

**FIGURE 10:**
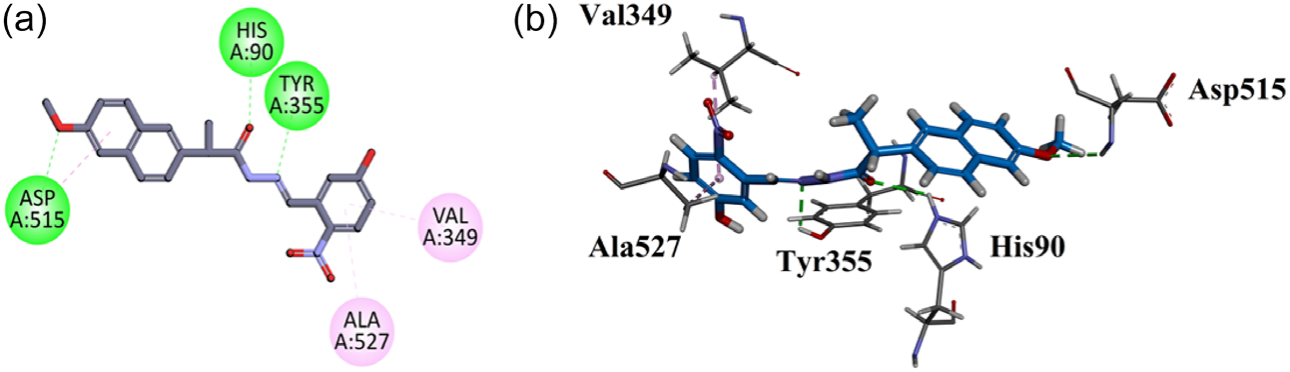
2D (a) and 3D (b) Plot of GMO‐I‐111a with COX‐2.

Compound **GMO‐I‐111a** has displayed 3 hydrogen bond hydrophilic interactions with the His90, Asp515, and Tyr355(residues interaction similar to native ligand). It also exhibited hydrophobic interactions with Val349 and Ala527 (Figure 10).

**FIGURE 11:**
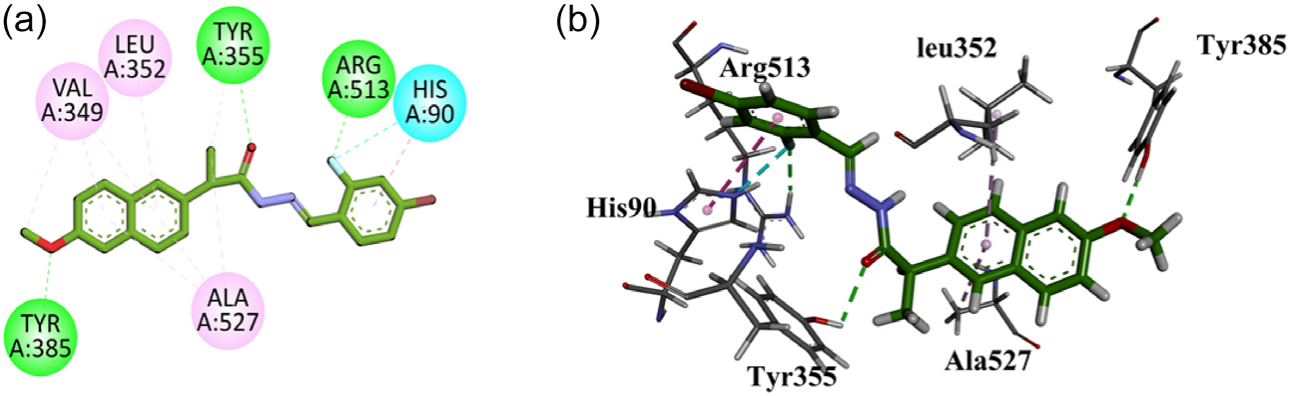
2D (a) and 3D (b) Plot of GMO‐I‐135a with COX‐2.

Compound **GMO‐I‐135a** exhibited three hydrophilic hydrogen bond (Tyr355, Arg513, and Tyr385) interactions similar to native ligands. Halogen binding affinity and π–π stacked interaction are displayed by His90. Other hydrophobic interactions (Leu352, Val349, and Ala527) include residues similar to native ligand residue interactions (Figure 11).

## 4 Discussion

Naproxen belongs to the propionic class of NSAIDs and is one of the analgesics used in overdose (11%) after ibuprofen (81%) [19]. The supratherapeutic dose changes the pharmacokinetics of this drug, i.e., prolonging the absorption and declining the peak plasma concentration [20]. The overdose causes upper gastric bleeding [21] and changes the liver function parameters [22]. Naproxen also prolongs the prothrombin time (PT) [21] and causes acidosis [22] in overdose. In addition to these toxic effects, this drug also has adverse actions on neurological processes and nephrons. These harmful effects might be managed clinically using various antagonistic substances, but medicinal chemistry will manage these ADRs by structure–activity relationship (SAR). In the current study, various naproxen derivatives were subjected to acute toxicity studies. The higher doses hindered animals’ feeding and drinking behaviors, indicating the bad effects on these related systems.

The biochemical test might be a good tool to compare the toxicity of naproxen and its derivatives. The acetic acid‐induced writhing‐induced test is one of the common peripheral analgesic models [5]. The pain induction by this chemical is attributed to the release of indigenous substances or mediators like arachidonic acid through stimulation of cyclooxygenase (COX) followed by biosynthesis of various prostaglandins (PGs). These PGs are then responsible for the induction of algesia, pyrexia, and inflammation [6]. These PGs might be accountable for the increasing peritoneal fluids and constrictions. These constrictions are observed in the form of abdominal contractions. The inhibition of these endogenous PGs will cause an analgesic effect.

In the current study, our tested naproxen derivatives significantly antagonized the induced writhes (*p* < 0.001). The analgesic effect in this model might be due to the inhibitor effect on COX by these derivatives. The parent drug (naproxen) blocks the COX‐I and COX‐II significantly [23]. The molecular basis of naproxen for interaction with COX is attributed to the *α*‐methyl and p‐methoxy groups present opposite on the naphthyl scaffold [7]. In the current study, all the naproxen derivatives have the mentioned substituents on the naphthyl scaffold. The tested compounds also resulted in significant antinociceptive in the thermal‐induced pain model. The thermal‐induced pain model is used to assess the central analgesic effect. The prolongation of latency time by tested compounds indicates the central analgesic effect. However, a detailed mechanistic study is recommended. The molecular docking studies also supported the antinociceptive effect of our tested naproxen derivatives.

## 5 Conclusion

It is concluded that tested naproxen derivatives are significant antinociceptive molecules. The COX‐I/II antagonist effect confirmed the nociception, followed by acetic acid and thermal induced pain model. In both of the pain paradigms, the compounds resulted in significant antinociceptive. In silico studies confirmed the targeted effect, too.

## Author Contributions

**Ubaidullah**: conceptualization (equal), data curation (lead), formal analysis (equal), investigation (lead), methodology (lead), software (equal), writing – original draft (equal). **Naveed Muhammad**: data curation (equal), formal analysis (equal), investigation (equal), methodology (equal), software (equal), writing – original draft (equal). **Abad Khan**: data curation (equal), formal analysis (equal), investigation (equal), methodology (equal), software (equal), writing – original draft (equal). **Rahaf Ajaj**: data curation (equal), formal analysis (equal), investigation (equal), methodology (equal), software (equal), writing – original draft (equal). **Abdur Rauf**: conceptualization (lead), project administration (lead), supervision (lead), writing – review & editing (lead). **Umer Rashid**: data curation (equal), formal analysis (equal), investigation (equal), methodology (equal), software (equal), writing – original draft (equal). **Fahad Hussain**: data curation (equal), formal analysis (equal), investigation (equal), methodology (equal), software (equal), writing – original draft (equal). **Rashid Khan**: data curation (equal), formal analysis (equal), investigation (equal), methodology (equal), software (equal), writing – original draft (equal). **Faiza Seraj**: data curation (equal), formal analysis (equal), investigation (equal), methodology (equal), software (equal), writing – original draft (equal). **Uzma Uzma**: data curation (equal), formal analysis (equal), investigation (equal), methodology (equal), software (equal), writing – original draft (equal). **Basharat Ali**: data curation (equal), formal analysis (equal), investigation (equal), methodology (equal), software (equal), writing – original draft (equal). **Abdul Sadiq**: data curation (equal), formal analysis (equal), investigation (equal), methodology (equal), software (equal), writing – original draft (equal). **Khalid** **Mohammed Khan**: conceptualization (lead), project administration (lead), supervision (lead), writing – review & editing (lead). **Marcello Iriti**: conceptualization (lead), supervision (lead), writing – review & editing (lead).

## Funding

This study was supported bythe Pakistan Academy of Science (Project No. 111).

## Conflicts of Interest

The authors declare no conflicts of interest.

## Data Availability

The data that support the findings of this study are available from the corresponding author upon reasonable request
